# Barriers to Cigarette Smoking Cessation in Pakistan: Evidence from Qualitative Analysis

**DOI:** 10.1155/2021/9592693

**Published:** 2021-11-08

**Authors:** Abdul Hameed, Daud Malik

**Affiliations:** Alternative Research Initiative (Pvt.) Ltd, Pakistan

## Abstract

**Background:**

With over 25 million tobacco users, Pakistan has one of the largest smoking populations in the world. Tobacco addiction comes with grave health consequences, especially for the poor and marginalized.

**Objective:**

This study explores barriers to smoking cessation in marginalized communities of Islamabad and the possibility of their use of Harm Reduction Products (HRPs), primarily e-cigarettes. *Methodology*. The study has used primary data of 48 respondents from marginalized communities. Several domains have been employed to evaluate the barriers to smoking cessation in these communities. Using qualitative technique, data was organized and categorized into objective themes.

**Conclusion:**

The experience of combustible smoking usually occurs in the 10-20 years' age bracket. Regular smokers in marginalized areas of Islamabad smoke 20 cigarettes or a pack per day. Their choice of cigarette brand is largely driven by affordability. Most smokers have made at least one attempt to quit smoking. Peer pressure and friendship are major barriers to smoking cessation. Lack of knowledge seems to be the major reason for not seeking medical assistance for quitting smoking. Knowledge about HRPs, especially e-cigarettes, can best be described as vague. Higher prices of the alternatives to combustible smoking are a major hurdle preventing their use for smoking cessation.

## 1. Introduction

Globally, tobacco is a major cause of more than 8 million deaths per year and a key risk factor for the development of multiple diseases, including lung, liver, oral, and throat cancers, Chronic Obstructive Pulmonary Disease (COPD), heart disease, and stroke [1]. A large proportion of these deaths—approximately 7 million—is a result of direct tobacco use while 1.2 million from exposure to secondhand smoke (SHS). The majority of smokers worldwide belong to low- and middle-income countries with different socioeconomic characteristics [2, 3]. Cigarette smoking increases the burden of disease and the probability of death. Historically, cigarette consumption has declined in various regions. Despite the reduced number of smokers in many countries, population growth continues to trigger an increase in cigarette consumption in China (0.71 trillion), Africa (0.03 trillion), the Eastern Mediterranean Region (0.09 trillion), and South East Asia (0.23 trillion). All these areas have distinct socioeconomic characteristics [4].

Pakistan, India, and Bangladesh are the most vulnerable countries, with a high proportion of consumers of tobacco and cigarettes [5]. Pakistan and Bangladesh are among those countries where a significant number of adults aged 15-65 years and older use tobacco [6]. Pakistan currently has an estimated more than 25 million tobacco users, and several types of tobacco products are available, including cigarettes, water pipes (“shisha”), stove, “gutka,” and “niswar” [7]. In Pakistan, smoking is a major cause of cardiovascular disease, lung cancer, emphysema, and chronic bronchitis [8].

Tobacco prevalence increases with age and decreases between the ages of 65 years and older in Pakistan. Smoking prevalence is highest in men aged 45 to 64 years. According to the Pakistan Demographic and Health Survey (PDHS), 23% men and 5% women used some form of tobacco in 2017-18, including cigarettes, “hookah,” “shisha,” “paan,” “gutka,” and “niswar.” The PDHS reports that 22% of men and 3% of women in fact smoke cigarettes. Pakistan has taken a number of initiatives within the framework of the WHO guidelines on tobacco control, including an increase in prices and taxation, enforcement of warning laws, bans on public smoking and advertising, and prohibition of sale of cigarettes in educational institutions. A price analysis of 20-stick packages of premium and cheapest cigarette brands in dollars in 2016 showed prices in Sri Lanka higher than in Pakistan, Bangladesh, and India. It is clear higher prices contribute to lower prevalence. Prevalence of cigarette smoking in Sri Lanka is less than in India, Pakistan and Bangladesh [9].

Various cigarette brands are available in Pakistan; they include Marlboro, Benson and Hedges, Dunhill, Gold Leaf, Capstan, Gold Flake, Embassy, Morven Gold, Diplomat, K2, Red and White, Gold Street Premium, and Kisan. According to previous study results, a typical 10% rise in the cost of 20-stick cigarette packets will reduce 4% of the adult cigarette demand [10]. A number of taxes were levied on cigarettes and tobacco products [11]. However, smoking cessation appears to be the weakest link in the fight against the tobacco epidemic in Pakistan. The success rate of smoking cessation is less than 3% [12]. Based on previous studies, most smokers in Pakistan want to quit smoking knowing that combustible smoking is cancer-causing and even acknowledge that SHS is harmful to the health of those around them and their families. However, even if they are aware of the dangers, they are unable to stop smoking [13, 14].

This qualitative study is the first one of its kind to highlight barriers to smoking cessation in marginalized, low-income communities. It assesses adult smokers' knowledge and understanding of the health hazards of smoking, as well as the critical question of why attempts to quit smoking remain unsuccessful. In particular, it examines the dichotomy between easy and cheap access to combustible tobacco and the lack of cessation services for marginalized communities. Therefore, a full understanding of the barriers to smoking cessation in marginalized communities will help to develop effective, indigenous, and accessible interventions.

This is perhaps the first study which has uniquely focused on the smokers in marginalized communities of Pakistan's capital vis-à-vis their smoking habits and quit attempts. All interviews were recorded in order to have a detailed picture of the respondent's smoking pattern and the quit attempts. Most of the interviews took place at the workplaces of the respondents. Another important focus was on the knowledge about cessation services through the perspective of socially and economically backward smokers. It highlighted the fact that the most ignored smokers in the marginalized communities have the most access to the unregistered, illicit, and the cheapest cigarette brands in Islamabad.  Section 2 of the study addresses material and methods. The findings of the empirical analysis are discussed in Section 3, while Section 4 focuses on discussion, and Section 5 reports policy implications and conclusion.

## 2. Material and Methods

### 2.1. Data and Instruments

**Figure 1:**
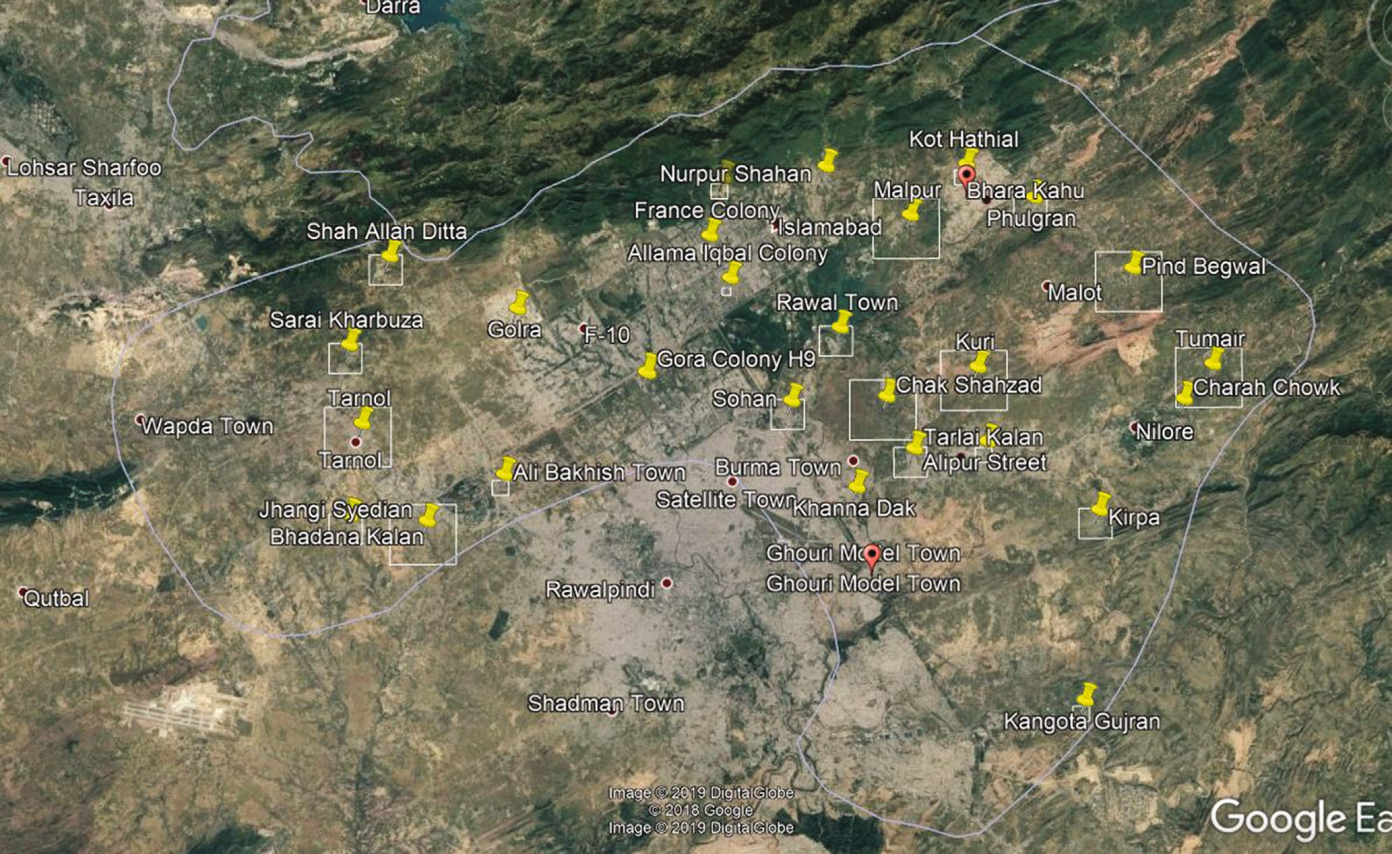
Study area map (Islamabad—capital of Pakistan).

The study has used primary data of eight areas out of 28 self-identified marginalized communities from Islamabad Capital Territory (ICT) of Pakistan (Figure 1).

Key Informant Interviews (KIIs) have been used for primary data collection. A semistructured questionnaire, prepared in English and translated into the local language (Urdu), was used for conducting KIIs. To verify the coherence and reliability of the questionnaire, it was retranslated from Urdu to English. To translate the KIIs questionnaire into Urdu, a specialized team of translators was formed. Two translators, who had no communication with each other, independently translated the questionnaire. A third senior translator reconciled the two versions to verify the final document was understandable and accurately conveyed the questions' substance. To ensure that the field teams accumulate and manage high-quality data, a two-day training session was conducted, with one day allocated for field practice session. The questionnaire was based on local and international literature on tobacco and smoking cessation. The study used Pencil and Paper Interview (PAPI) and Digital Voice Recording (DVR) for primary data collection, which is a simple and precise data collection technique with high-quality results and high precision. Furthermore, DVR was turned into transcripts based on study themes. This procedure was overseen closely by the survey project manager and senior research analyst, who ensured the substance of the questions was clearly and correctly conveyed in the translated scripts. This was done to verify that the translation properly conveyed the respondents' views.

### 2.2. Sampling

A two-step sampling for the selection of respondents employed a self-constructed frame. Qualitative research requires a smaller sample for measuring and exploring goals and scope, compared to quantitative research. Qualitative samples must be large enough to obtain enough data to adequately describe the research objectives. In other words, qualitative research is achieving optimal saturation. With respect to qualitative data, [17] suggested 30 to 50 interviews, while [18] suggested only 20 to 30. This study was conducted with 48 KIIs, which is enough to achieve saturation.

### 2.3. Selection Procedure

During the first step of determining the Primary Sampling Units (PSUs), 14 urban and rural charges (A charge is a census defined geographical area used in the 2017 population census in Pakistan. Each charge has several circles with each circle comprising several census blocks (enumeration areas): http://www.statistics.gov.pk/assets/publications/Pakistan%20Paopulation%20and%20Housing%20Census-2017%20National%20Report.pdf) in the Islamabad district were divided into 28 self-identified marginalized areas (Figure 1), followed by a random collection of eight marginalized areas. In the second step, a Quick Count listing of at least 40-50 potential individuals (who had firsthand knowledge about barriers to smoking cessation and use of HRPs) in the target population was used in each selected PSU. The criterion for the respondent selection was as follows:

1. 18 years and above
2. Adult smoker residing in the marginalized community area

Furthermore, based on each selected PSU and the list of potential individuals, the required number of diverse individuals was selected using simple random sampling (Table 1).

### 2.4. Methodological Framework

**Figure 2:**
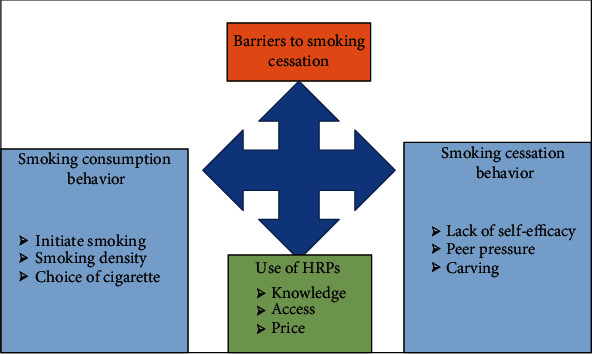
Conceptual framework.

The study used several domains to evaluate barriers to smoking cessation in the marginalized communities of Islamabad. These included, in particular, the demographic and socioeconomic background of the smoker, smoking and quitting behavior, factors that may convince a smoker to quit smoking, the possible use of HRPs to quit smoking, and assessment of individual perceptions regarding smoking cessation policy.  Figure 2 depicts the conceptual framework, which used qualitative techniques to analyze obstacles to smoking cessation in Pakistan. This study included three primary themes linked to smoking consumption and quitting behavior, as well as the usage of HRPs to quit smoking. Furthermore, these overarching themes have been subdivided into subthemes. The study evaluated four domains that might impact smoking cessation as policy predictors:

1. Individual-level control of combustible smoking, for example, use of cigarettes, health-related risk, social and family life constrictions, and well-being
2. Community and indoor workplace level control of smoking, for example, prohibited smoking at workplace
3. Smoking control regulation and guidelines
4. The possibility of using HRPs to quit smoking

Internal validity, dependability, objectivity, and external validity are common concepts used by quantitative researchers. This study has adopted several steps that evaluated Lincoln and Guba's fundamental Four-Dimension Criteria (FDC) to generalizability, internal validity, dependability, and objectivity. The trustworthiness relates to how qualitative research ensures credibility, reliability, conformability, and transferability [30]. The following steps have helped to assess the investigator's confidence in the reality of findings based on the research design, informants, and context:

1. Credibility: to establish confidence that the results are true, credible, and believable, this study employed simple random sampling to select potential respondents. Further, to ensure field teams are capable of accumulating and managing high-quality data, a two-day training session was held. Additionally, one day was allocated for field practice for enumeration and data collection. This study also used Digital Voice Recording (DVR) for primary data collection, which is a simple and precise data collection technique with high-quality results and precision
2. Dependability: to ensure the findings are repeatable if the inquiry occurred within the same cohort of participants, coders, and context, this study used detailed drafts of the study protocol, including semistructured questionnaire based on the previous literature, translation into local language, training of supervisor and enumerators, and data analysis plan. Additionally, a detailed track record of the data collection process and stepwise data coding was employed to convert information into themes
3. Conformability: to extend the confidence that the results would be confirmed or corroborated by other researchers, this study employed line-coding for open-ended questions and key concepts—statements moved to subcategories and broken down into conceptual components and indicators to make sense of data. Moreover, relation and causal links have been built between categories using STATA software
4. Transferability: to extend the degree to which the results can be generalized or transferred to other contexts or settings, the study used simple random sampling, instead of purpose sampling. Simple random sampling produced self-weighted proportion or prevalence of research indicators. Quantified operational and theoretical data saturation in discussion section with literature verification

### 2.5. Data Analysis

In qualitative research, data analysis is a systematic process of examining and organizing qualitative information in the form of interview transcripts, observation notes, or other nontextual resources. Evaluating qualitative data entails coding or classifying the data. Essentially, it is to make sense of massive data by decreasing the volume of raw information, detecting relevant patterns, deriving meaning from data, and lastly creating a logical chain of evidence [29, 31]. The study has used stepwise process of analysis to evaluate qualitative information.

1. Data collection and transcribed to text: qualitative information has been turned into transcripts based on study objectives. This procedure was overseen closely by the survey project manager and senior research analyst, who ensured the real meaning of questions was clearly and correctly conveyed in the translated scripts. This was done to verify the translation properly conveyed the respondents' views. Create themes: qualitative transcribed text data divided into study themes
2. Developed categories: prepared data categories in accordance with themes and subthemes
3. Data coding and synthesis: used line-coding for open-ended questions and key concepts. Moved to subcategories, statements were broken down into conceptual components and indicators to make sense of data. Moreover, relation and causal links have been built between categories

### 2.6. Ethical Consideration

The study was approved by the ARI internal Ethics and Technical Committee to ensure research quality and ethics. A verbal consent of the participants was obtained before starting the interview. Furthermore, confidentiality, anonymity, and honesty follow from this premise.

## 3. Results

### 3.1. Respondent Characteristics

The findings show that of the 48 respondents, 15% were between 18 and 24 years of age, 54% were aged between 25 and 44 years, and 31% were 45 to 64 years old. Education levels were classified into six groups—bachelors or master's, intermediate (Fsc/FA/A levels), matriculation (10^th^ grade), middle (8^th^ grade), primary, and illiterate. The majority (69.%) of the respondents had schooling up to matriculation (10th year), with only 8% going beyond the 10^th^ year (intermediate, bachelors, and masters). At least 15% of the respondents were illiterate. Furthermore, 46% were employed, 48% were self-employed, and 6% were unemployed. Employment status shows combustible smoking is much more common among self-employed workers than the employed and the unemployed. However, the proportional difference between salaried smokers and self-employed smokers was small but statistically significant between self-employed smokers and unemployed smokers.

The personal income of combustible cigarette smokers was classified into four groups—less than and equal to 10k, 11k-20k, 21k-30k, and more than 30k. To this effect, 8% combustible cigarette smokers earned an average monthly income of less than Rs. 10,000 ($67), 44% earned Rs. 11,000-20,000 ($73-133), 33% earned Rs. 21,000-30,000 ($140-200), and 15% earned a monthly income of more than Rs. 30,000. These income estimates indicate the majority of combustible cigarette smokers have an average personal income of between Rs. 11,000 and 20,000 (see Table 2).

### 3.2. Smoking Consumption Behavior

In marginalized communities of Islamabad, men have a higher chance of smoking their first cigarette before the age of 18 years. Most respondents reported their first experience with combustible cigarettes between the age of 10 and 15 years. One respondent reported having initiated smoking when he was “in grade 2 or 3.” As he would play with an older friend who used to smoke, “*we would smoke a bit. Afterwards, I would steal one or two sticks from my father's cigarette pack. So you can say that I started proper smoking when I was in fifth grade*.”

#### 3.2.1. Reasons to Initiate Smoking

The primary reason for initiating smoking (age range 10-20 years) is the company of and friendship with smokers within and outside the household, and at the workplace. An environment where smoking is accepted as normal social behavior by seniors and friends entices young people to start smoking as teenagers. It is considered part of everyday life, with no social stigmatization attached. Those with friends or family who smoke are more likely to initiate smoking than those without.

When in their teens, the curiosity of trying out smoking just for the fun of it is a major reason for becoming a smoker—60% of smokers attributed their smoking initiation to friends and fun. This indicates the company of friends who are smokers is a strong pull for initiating smoking. The respondents recalled that when they *saw their friends smoking, they also started smoking.* The use of a tobacco product in the household as a normal social practice leads to initiation of smoking. A respondent recalled, “*When I was a child, my grandmother used to smoke ‘hukka' (water pipe). It was my responsibility after coming from school to fill the water pipe with tobacco. While performing that duty, I would also have one or two puffs of the water pipe. Of course, afterwards I also started smoking cigarettes.*”

In the workplace, the presence of smokers is an important reason for smoking initiation. The combination of economic pressure and smoking company is too strong to withstand. One respondent argued that when he started looking for work after the death of his father, “*most of the people I met were smokers.*” He also began to smoke as a normal social behavior. In marginalized communities, smoking is seen as providing relief from stress caused by a limited economic situation.

One respondent said since he was poor and depressed, he took to smoking to relieve stress. “*Now it is a habit, which is very difficult to give up.*” In the marginalized communities of Islamabad, a regular smoker consumes 20 cigarettes or a pack per day, which is more cigarettes per day than the national level.

#### 3.2.2. Smoking Density

In this study, we asked respondents when and why they smoke more than usual. Overall, more than two-thirds of respondents reported smoking more cigarettes than usual. Tension is the main reason for smokers to consume more cigarettes than their average consumption. Mostly when worried, smokers invariably smoke more. While for others, as smoking becomes a habit, it becomes an essential part of daily life. They may smoke more in the morning and after lunch. Some said their cigarette consumption increases during winters. Others reported when in the company of friends who are also smokers, they consume more cigarettes than usual. Similarly, some smokers when busy in a task may smoke more than their usual quota of cigarettes.

#### 3.2.3. Choice of Cigarette Brand

For smokers in marginalized communities, the choice of cigarette brand is largely driven by affordability. A little more than half of the respondents (54.2%) opted for Capstan, mainly because it is cheap. A pack of Capstan costs less than half a dollar in Pakistan. The possibility of changing brands, depending on the income of the respondent in the marginalized communities, is frequent. One of the respondents currently using Capstan said he would look for local cheaper alternatives. These include locally made unregistered and tax-evading cigarette brands such as Kisan.

### 3.3. Smoking Cessation Behavior

The study found that most smokers (75%) made at least one attempt to quit smoking, but these attempts were made without any medical assistance. Even though the respondents made several attempts, they were unable to stop smoking. While quitting smoking is urgently needed, attempts to quit smoking are not successful. This was pointed out by one respondent who tried to stop smoking every two months before reoffending.

#### 3.3.1. Barriers to Smoking Cessation

Barriers to smoking cessation were derived from self-reported reasons and causes for smoking behaviors among respondents. Most smokers reported having made attempts to quit but failed. While they recognize smoking as a health hazard, they continue to do so based on their individual beliefs, priorities, and lack of knowledge and medical assistance. Most attempts to quit smoking in Pakistan are made without help.

#### 3.3.2. Lack of Self-Efficacy

Self-efficacy is conceptualized as self-control or belief in our ability to overcome given challenges and successfully complete tasks. Since respondents have been unable to quit smoking despite several attempts, they try to justify the failure with two diametrically opposed attitudes—helplessness in giving up smoking and the expression of confidence in their strong will to quit as and when they wanted. One of the study participants said he did not have the will power to quit while another was confident that he would be able to quit whenever he so decided. Others said the habit of smoking is too strong to quit.

### 3.4. Physiological Barriers to Cessation

At the individual level, physiological factors such as tension, stress, and headache are common among smokers. One participant said worries turn him towards smoking. Another identified numerous reasons for not quitting smoking; these included tension, stress, headache, and poverty. However, a participant saw companionship in smoking, saying, “*When one is alone, what should one do but smoke*. *Cigarette is your companion in loneliness.*”

According to literature, prolonged smoking leads to stress, tension, and headache. Conversely, smokers find tobacco as a source of relief from these symptoms. In the long run, it becomes a habit and causes stress, anxiety, and tension.

#### 3.4.1. Peer Pressure

Peer pressure is a major barrier to smoking cessation. It is important to highlight that the environment in which smoking is accepted as normal social behavior works both ways—as an attractive and accepted invitation to initiating smoking and as a strong barrier to cessation. Friends, family members, school and college fellows, and colleagues play a significant role in influencing decisions made by an individual. The participants' inability to resist peer pressure—the company of smoker friends—remains a strong barrier to quitting smoking. One of the participants narrated the difficulty in saying “no” to smoker friends.

“If you have friends who are smokers, it is very, very difficult to give up smoking. When you are with them, you are bound to smoke.”

Mostly, respondents reported close friends and the surrounding environment as a barrier.

“It has happened more than often that just when I am in one of those quit smoking periods, I meet a smoker friend, and before I know, I start smoking again.”

Some respondents understood that smoking brings no relief from tension and worries but pointed out that cessation is a difficult task in an environment in which smoking is an accepted behavior.

“People think smoking brings some kind of relief, such as you forget your worries. I do not think that is the case. Peer pressure is a major hurdle in smoking cessation. Your surroundings are most critical to your attempt to quit. When you are among smokers, you will inevitably start smoking sooner or later. Even a non-smoker will start smoking.”

#### 3.4.2. Craving

Some of the respondents said craving for the habit of smoking is a barrier. They said the habit of holding something in their hand, especially when they are alone, is too strong to resist. Even the real-life experiences of how combustible smoking results in serious health problems fail to convince them to quit.

“One of my cousins in Lahore fell ill because of smoking. He was admitted to a hospital for heart disease. I saw his condition, got scared, and decided to quit smoking. For a brief period, I thought I too could fall ill because of smoking. But I could not quit smoking because of its craving. You know your hands need something to hold and smoke. Your hands grow used to holding a cigarette.”

### 3.5. HRPs and Marginalized Communities

In Pakistan, e-cigarettes are legally imported and sold. In this sample study, the current knowledge about HRPs, especially e-cigarettes, can best be described as vague in Islamabad's marginalized communities. Only one-third of the respondents knew about HRPs. It is important to highlight that e-cigarettes are the only HRP they know about. None of the respondents, it seems, used HRPs with the intent of smoking cessation. Those who used e-cigarettes did so more out of curiosity than anything else. There was no evidence of any respondent opting for a longer use of e-cigarettes with the intent of harm reduction or smoking cessation. Friends are the main source of knowledge about HRPs. This also shows members of marginalized communities may come to know about HRPs but they seem uninterested in buying, largely because of high prices.

An e-cigarette device in Pakistan costs Rs. 3,000-18,000 ($20-120). The expenditure on e-liquids makes e-cigarettes costlier. Most of the respondents smoked local cigarette brands which cost less than Rs. 2,100 ($14) a month. The respondents who said they have used an e-cigarette took it from their friends. Only one respondent said he bought an e-cigarette. Additionally, the respondents (56%) who have used an e-cigarette have no idea about their prices. This is mainly because they took e-cigarette from their friends. It is evident that higher prices of alternatives to combustible smoking are a major hurdle to their use for smoking cessation or as a harm reduction product. One of the respondents shared his experience of using nicotine gum as a smoking cessation tool. However, he found the nicotine gum expensive. A pack of nicotine gum costing Rs. 800 ($5.3) was too expensive for the respondent. However, he continued to smoke combustible cigarettes alongside using nicotine gum. Though there is vagueness about HRPs, most of the respondents expressed readiness to use e-cigarettes with the intent of smoking cessation or harm reduction. However, they want the prices of HRPs to be heavily subsidized.

## 4. Discussions

In marginalized communities, it is highly likely that smoking initiation will begin before the age of 18 years. This can be due to the presence of older smokers at home (fathers, uncles, brothers, etc.), no parental guidance or monitoring, lack of knowledge about the legal age to start smoking, and poor enforcement of tobacco legislation. Less educated or illiterate populations have high smoking prevalence, as less educated smokers find it more difficult to quit smoking [19]. Recent research shows that lower level of education and poverty or social deprivation are also associated with higher rates of smoking [20]. Moreover, peer pressure is also a major obstacle to smoking cessation in marginalized communities. Critically, the environment in which smoking is an accepted social behavior works as an attractive and accepted invitation to initiating smoking and as a strong barrier to giving it up. The question arises as to why smoking cessation or quitting is not effective in Pakistan. Pakistan's lack of quit-smoking services, alternative nicotine delivery systems (ANDs), and policy-based research on the barriers to combustible smokers are key fences [15, 16].

The main reason for starting smoking is the company and friendship of smokers within and outside the household, and at the workplace. The environment in which smoking is a normal social behavior leads to young people initiating smoking. The curiosity of trying out smoking just for the fun of it is a major reason for a teen becoming a smoker. In this study, most of the smokers have made at least one attempt to quit smoking. However, these attempts have been made without any medical help. Most of the quitting attempts in Pakistan are made without assistance. Exposure to secondhand smoke is a serious health concern in Pakistan. More than half of the nonsmoking adults (56%) and one-third (34%) of youth (13-15 years) are exposed to SHS in public places [21].

The study participants were not aware about the presence of smoking cessation clinics in Islamabad or elsewhere in Pakistan. Some of them, for the first time in their lives, have come to know about a smoking cessation clinic. Literature points to a strong relationship between health risk and cigarette consumption. In many studies, the lowest cigarette consumption bench was set at 1-9 or 1-15 cigarettes per day, investigating communicable, heart, and lung-related diseases [22, 23]. However, heavy smoking can lead to schizophrenia [24]. Since marginalized communities in Pakistan lack access to health facilities, their deficient knowledge about smoking cessation clinics is understandable. Further, lack of knowledge about the health hazards of smoking seems to be the major reason for not seeking medical assistance for quitting smoking. Respondents in marginalized communities did not consider smoking a health issue and therefore did not feel the need to consult a doctor in this regard.

As none of the respondents has been able to quit smoking despite making numerous attempts, they try to justify the failure in two diametrically opposed attitudes—helplessness in giving up smoking and the expression of confidence in their strong will to quit as and when they want. For most of the KIIs, an increase in the prices of cigarette packs would force them to look for cheaper alternatives. The availability of cheaper and illicit cigarette brands is a major issue in Pakistan. As cigarette prices in Pakistan are the cheapest in the world [7], the cheaper options for smokers in the marginalized communities are multiple. Though none of the respondents has succeeded in quitting smoking, most seek help in this regard. They want smoking cessation clinics at health facilities. For the understanding of smoking cessation behavior, most studies have used daily smoking amount of nicotine or number of cigarettes for assessing quit attempts, quit success, and use of cessation assistance [25]. The annual success rate of quitting smoking in Pakistan is only 2.6% [13]. Though every year around 25% of smokers make an attempt to quit smoking in Pakistan, 97.4% fail to quit [14]. Numerous experimental studies observed main reasons behind multiple efforts along with high failure rate are lack of clinical and health care delivery systems, effective treatments, practical counseling, and social support [25].

Current knowledge about HRPs, especially e-cigarettes, in the marginalized communities of Islamabad can best be described as vague. None of the respondents has used HRPs with the intent of smoking cessation. Those who used e-cigarettes did so more out of curiosity than anything else. According to the proponents, e-cigarettes are 95% less harmful than conventional or combustible tobacco [26, 27] and it is useful for quitting smoking [28].

There was no evidence of any respondent opting for prolonged use of e-cigarettes with the intent of harm reduction or cessation. Friends are the main source of knowledge about HRPs. Respondents who used an e-cigarette took it from their friends. Members of the marginalized communities may know about HRPs, but they seem uninterested in buying them, largely because of high prices [32]. An e-cigarette device in Pakistan costs Rs. 3,000-18,000 ($20-120). The expenditure on e-liquids makes e-cigarettes costlier. Most of the respondents are smoking local cigarette brands which cost less than Rs. 2,100 ($14) a month.

## 5. Conclusion and Recommendations

This study explores barriers to smoking cessation in marginalized communities in Islamabad and the possibility of using HRPs. In the marginalized communities, the first combustible smoking experience usually occurs between 10 and 18 years' age bracket. The main reason for initiating smoking is the company and friendship of smokers within and outside the household, and at the workplace. Smokers in these communities are consuming more cigarettes per day than the national level. On average, a regular smoker in marginalized areas in Islamabad smokes 20 cigarettes or a pack per day. Respondents reported stress as the main reason for consuming cigarettes more than their average consumption. Their choice of cigarette brand is largely driven by affordability. They would opt for the least expensive legally sold brand in Pakistan. A little more than half of the respondents opted for Capstan, mainly because it is cheap. In this sample study, most of the smokers have made at least one attempt to quit smoking. However, these attempts have been made without any medical help. Peer pressure is a major barrier to smoking cessation. Lack of knowledge seems to be the major reason for not seeking medical assistance for quitting smoking. Knowledge about HRPs, especially e-cigarettes, can best be described as vague. Friends are the main source of knowledge about HRPs. Higher prices of alternatives to combustible smoking are a major hurdle to their use for smoking cessation. Smoking cessation mechanisms are missing from tobacco control efforts in Pakistan, especially for marginalized communities. Evidently, smokers in marginalized communities need help in quitting smoking. There is a need to establish smoking cessation clinics in hospitals and create buy-in about them through mass awareness. The main barriers to quitting smoking are lack of medical and clinical assistance, peer pressure, and low perceived risks of smoking. There is a need to provide medical and clinical assistance for quitting smoking. This assistance should be backed with public advocacy on the negative effects of combustible smoking. Easy availability of cheap smoking options is a major barrier to smoking cessation. Lack of tobacco-control law enforcement, especially in marginalized areas, is the other demand side barrier. Tobacco law enforcement on smoking at public and private places should be ensured. Lack of knowledge about alternatives (HRPs) to combustible smoking and their higher prices in Pakistan is a barrier to their adoption. There is a need to create an understanding about HRPs, backed by sensible regulation.

## 6. Limitations and Further Research

The study has been limited by several constraints. It used a qualitative design instead of using prevalence significance. Therefore, the sample population is not fully represented at the national level. Interviewing women in Pakistan is difficult due to cultural constraints, especially among tobacco users. Women do smoke in Pakistan but avoid smoking in the public, and additionally, they would avoid discussing their smoking habit. There, we were unable to find an adult female smoker. There is a need for national and provincial level research to assess barriers to smoking cessation in marginalized communities in Pakistan and the possibility of using HRPs.

## Figures and Tables

**Table 1:** Sample size.

Selected marginalized areas (PSUs)	Number of listed individuals	Selected individuals
Bhara Kahu	45	6
France Colony, F-7	42	6
Ghauri Town	49	6
Golra Sharif	49	6
Gora Colony (Rimsha Colony)	46	6
Saidpur Village	51	6
Tarlai	50	6
Tarnol	50	6
Total	382	48

**Table 2:** Respondent characteristics.

Demography characteristics of participants (%) *n* = 48		
Gender	Male	100.00
Age in years	18-24	15
	25-44	54
	45-64	31
Education	Illiterate	15
	Primary	27
	Middle	15
	Secondary	27
	Higher secondary	8
	Bachelor and master	8
Employment	Unemployed	6
	Self-employed	48
	Employed	46
Monthly personal income (Rs.)	Less ≤ 10k	8
	11k-20k	44
	21k-30k	33
	30k≥	15

## Data Availability

The data can be obtained from the corresponding author upon request.
